# Remarks on the Cause and Treatment of Cholera Infantum

**Published:** 1853-08

**Authors:** L. P. Yandell

**Affiliations:** Professor of Physiology and Pathological Anatomy in the University of Louisville; Louisville


### WESTERN JOURNAL

OF

### MEDICINE AND SURGERY.

---

AUGUST, 1853.

---

### ART. I.—*Remarks on the Cause and Treatment of Cholera Infantum.* By L. P. YANDELL, M. D., Professor of Physiology and Pathological Anatomy in the University of Louisville.

Cholera Infantum has attracted much of the attention of medical writers in our country, as it seems, indeed, to be a disease almost peculiar to the climate of the United States. In 1794, Dr. Rush published an interesting account of the disease, which was called in Philadelphia the "vomiting and purging of children," and in Charleston, "the April and May disorder." The description of the complaint by Dr. Rush* is one of the most graphic and faithful ever given. Dr. Edward Miller, one of the editors of the *Medical Repository*, published in the first volume of that work a valuable article on the same subject—

---

* See *Medical Inquiries*, voi. 1, p. 159, 1st Ed.

"the cholera or bilious diarrhœa of infants," as he styled the affection. The medical journals of our country have abounded in dissertations on the subject since. It is proposed to add one more to the number; for although cholera infantum is by no means the fatal disease it was twenty-five years ago, it is still a complaint much dreaded, a source of great suffering, and of a fearful mortality among children.

It is the observation of physicians who have been long engaged in practice, that the "summer complaint of infants" has greatly abated within the period referred to. It seems to have been on the decline since the disappearance of epidemic cholera from our country, in 1835, and has certainly been much less prevalent during the last ten or fifteen years. The circumstance is an interesting one, that in the same period bilious remittent fever has prevailed far less extensively than it did twenty-five years before. Within the last fifteen years we have seen this fever, to a very considerable extent, superseded by typhoid fever. Next to bilious remittent fever, Dr. Harrison remarked, in 1828, cholera infantum required most the attention of Louisville practitioners; and before that time Dr. Meigs had stated, that one-third of the deaths among children in Philadelphia were occasioned by this disease alone. Dr. Joseph Parrish declared, in 1826, that "no disease contributed so largely to swell the bills of mortality in large cities, during its prevalence," as the one under consideration. "Were it not restricted to the summer season," he continues, "it would prove a greater scourge to the community than consumption itself."*

The fact of the decline of cholera infantum with bilious fever, doubtless has its significance. The relationship of the two diseases was early recognised. Dr. Rush observed that a fever of the "remitting kind," with "evident

* North American Med. and Surg. Jour. vol. 2, p. 68.

exacerbations, especially in the evening," attended upon cases of cholera infantum. Cleghorn, in his work on "Diseases of Minorca," had previously made the same observation. Dr. Edward Miller regarded the cholera of infants and bilious fever as branches of a common stock. But this affinity has not been universally admitted by medical authors. Dr. Parrish, in the paper referred to, ascribes cholera infantum to the agency of heat alone, unaided by miasms; and not a few writers have attributed it, with the common people, to worms, summer fruits, and dentition.

The question is a practical one. Our conception of the etiology of the disease will necessarily influence our method of cure. If there be a real connection between the complaint and malaria, it is important to be assured of the fact. This is the chief point of the present paper; and it is esteemed one of moment for the reason, that, if established, it must lead to a more efficacious method of treating cholera infantum.

Diarrhœa in children, there can be no doubt, is frequently excited by teething, and by indigestible food, whether fruits or other articles, just as similar causes give rise to disordered bowels in adults. Such disorders may be very severe, destroying life in a few hours, as cholera morbus is known to do; but most generally they are mild, and may be cured by an opiate, or a single dose of calomel. They, probably, do not come under the head of cholera infantum; they are unattended by fever, and do not recur after the patient has once been relieved. They are liable to occur at all seasons and in all places. Cholera infantum is eminently a relapsing disease. "We may obviate the more violent symptoms," says Dr. Parrish,* "we may procure temporary relief; we may even flatter ourselves that a cure has been effected; but the original causes have not lost their power; an increased susceptibility to their

* Op. cit. p. 68.

operation remains; relapse upon relapse is experienced; and at last the little sufferer, worn out by the successive attacks, sinks beyond the reach of medicine and expires."

The impression prevailed with some of the earlier writers on cholera infantum, that it is especially a disease of cities; and hence the advice of Dr. Rush and of Dr. Parrish to the parents of the little patients, to remove them, if possible, to the country. But it is well known at the present time, that children in some parts of the country suffer with the complaint as severely as do those in the city. Where benefit was derived from change of air, it was in cases where sufferers were taken from a malarious to a healthy atmosphere. This was the case in the neighborhood of Philadelphia; but the environs of our city are less healthy than the city itself, and no such beneficial effects are experienced here from the removal of sick children to the country. Patients with intermittent fever often get well on removing to a pure atmosphere.

Heat is generally regarded as the efficient cause of cholera infantum; the common name for the disease is "the *summer complaint.*" Dr. Rush affirms that "its frequency and danger are always in proportion to the heat of the weather." Dr. Harrison makes the same statement. Is it true, that there is this exact correspondence between the complaint and the temperature of the weather? The fact that the disease has been declining for several years, while the summers have been as hot as ever they were, disproves such a supposition. The experience of the present summer is full in the face of it; we never passed through so hot a June in this region of Kentucky, and, on all sides, it is admitted, that there was never less cholera known among infants in that month, than during the last. "Although it is unquestionably true," says Dr. Cooke,* "that hot weather is the season in which cholera infantum is epidemic, it is to be observed that this disease

* Transylvania Journal, vol, 1, p. 192, 1828.

is not always most prevalent in the hottest summers. The summer of 1821 was, in the upper part of Fauquier county in Virginia, after July, uncommonly hot and dry, insomuch that the grass and even the Indian corn was burnt up; it was, nevertheless, uncommonly healthy." Cleghorn, in the work already quoted, records a similar observation. The weather in Minorca had seldom been felt so excessively hot as in the summer of 1749, and the drought was such that the farmers, in some parts, "scarcely reaped as much corn as they had sown." But notwithstanding the excessive heat, he says, "in June and July there were some specimens of the summer diseases, but so few that they scarcely deserve to be called epidemical."

Heat alone, then, we may conclude, will not develope cholera infantum. When the hot weather was attended with rain, Cleghorn encountered cholera enough, and the "wettest," he says, "are the most sickly seasons." At the same time, we have to admit, the prevalence of this complaint does not appear to be governed entirely by the weather, for we have seen that while our seasons remain the same the disease with us has been for some time declining. All that seems to be fully made out is, that the circumstances under which bilious remittent and intermittent fever prevail are those which favor the prevalence of cholera infantum—that they belong to the same family of diseases. This is all that is important in the present inquiry.

If, as Dr. Edward Miller suggested, "the cholera of infants forms one branch of that large stock of diseases" of which remittent and intermittent fevers are another type, it follows that the treatment should conform to the same principles. But it has not done so. The therapeutics have not had proper reference to the etiology of the disorder. The minds of physicians have been quite too much occupied with a single symptom of the disease—the diarrhœa. Our remedies have been too exclusively directed to the disordered state of the stomach and bowels.

Dr. Miller, in the paper more than once alluded to, says, that one of the principal objects of his communication was to recommend "the trial of mercury, alone or combined with opium"—a practice suggested by the work of Clark on "Diseases of Hot Climates." It succeeded so completely, he remarks, in a case of infantile cholera, attended with dysenteric symptoms, that he soon extended the use of calomel to the bilious diarrhœa of children, and found it to answer as happily as before. Some of his medical friends made trial of it, and, as they assured him, "with singular advantage." From this time forward the mercurial treatment became popular in cholera infantum; and its efficacy has unquestionably been great. The experience of the profession is probably not more uniform as to any point, than the relief afforded by mercurial purging in cholera and diarrhœa. The substitution of bilious evacuations for the watery discharges that attend these diseases, is well known to afford the greatest relief. Children laboring under the summer complaint rarely fail to be benefitted by bilious purging. So soon as the character of their passages is changed, there is an improvement in all their symptoms. Calomel checks the disease, but too often fails to cure it, and it returns again and again.

In one of these relapsing cases, in the summer of 1841, I thought I observed a connection between the return of the diarrhœa and evening exacerbations of fever, and was led to prescribe sulphate of quinine with reference to the chill which was supposed to precede the fever. The effect was most salutary, and the little patient was soon effectually relieved of his troublesome disorder. Other cases have been treated since in the same way, and with results as satisfactory. Dr. Booth has lately recommended the practice, in an article published in the last volume of this Journal, having called the attention of the profession to it some years ago in the New York Medical Gazette. My conviction is that the practice is valuable. Doubtless there are cases of bowel complaints in infants which do not call for this remedy; but in many, it is believed, periodicity will be found to characterize the disease, and in these there can be no question, that quinine is *the* remedy.

Children often suffer excessively in this disease from being denied cooling drinks. The thirst is intolerable; but from prejudice, or because the sufferer is not able to speak and make its wants known, water is not given. A few weeks ago, I saw a child affected with the complaint whose cries satisfied me that it was suffering from thirst. Its mother said that the same plaint had been kept up throughout the preceding night. Water was given, and it drank eagerly As its stomach was not disordered, the child was permitted to drink as much as it wanted, and its cries ceased. The diarrhœa was easily arrested by tincture of opium; quinine was administered, and the patient remains free from the the disorder.

In an other case, some years ago, the thirst was accompanied by high fever, and an irritable stomach. The water which was freely allowed, was vomited almost as soon as it was swallowed. This patient was immersed in a cool bath, and with the fever, which soon subsided, he was relieved of his nausea and tormenting thirst. In very many cases, ice has been prescribed when the stomach would not retain water, and with effects as gratifying as any that have ever been presented to the writer in the practice of physic. The cold bath, ice, and cold drinks, constitute remedies of inestimable value in the treatment of this complaint. It is singular that they are not in more universal use. Dr. Miller urged them strongly in his excellent paper, published nearly fifty years ago. "As the cholera of children," he says, "is a febrile disease, and the surface of the body often heated far beyond the proper point, it will be advisable to expose all such parts of the skin as feel too warm to the hand, to a stream of cool air or *to bathe them in cool water*. Several times a day," he continues, "the patient should be washed in vinegar and water, salt and water, or water alone, by means of a sponge, as he lies in bed, with as little motion, disturbance, or fatigue, as possible." Dr. Dewees wrote, twenty-seven years ago—"We have several times seen the most prompt advantage result from exhibiting to the little sufferer very small pieces of ice, every few minutes. It seems more certainly to abate thirst, and at the same time tranquillize the stomach better than water."

Every physician who has ever made trial of this practice has witnessed the same result. The effect is always salutary. The strong demands of nature, and true therapeutics are here, at least, at one. That which the sufferer craves above all other things, is that which more than al other things besides will contribute to his relief. There are cases in which cold enemata, introduced very gently, exert a happy influence. These are such as show a dysenteric tendency, and are accompanied by much febrile reaction.

Although the opinion has been expressed in this paper, that the diet of children alone will not induce cholera infantum, their diet and drink are by no means matters o secondary importance. They may become the exciting causes of the disorder. There can be no question that they are continually provoking disease in the digestive organs of the young, even more than of the old. The quality of the water affects the health of infants in an unequivocal manner. It is the united testimony of the profession of Nashville, that since the erection of water-works there the children of that city have suffered much less with bowel complaints, and the improvement is attributed to the substitution of river for limestone water.

But it was not my purpose to enter into details, whether as to the prevention or treatment of cholera infantum.

It has appeared to me that the *febrile* character of the disease has been too much overlooked, and that consequently remedies of the most undoubted efficacy in fevers have not had a fair trial in the cholera of infants. Under this impression these pages have been hastily written, and are now respectfully submitted to the profession.

Louisville, July 12th.

